# A Multifunctional Conjugated Polymer Developed as an Efficient System for Differentiation of SH-SY5Y Tumour Cells

**DOI:** 10.3390/polym14204329

**Published:** 2022-10-14

**Authors:** Angelo Nicosia, Giuseppe La Perna, Lorena Maria Cucci, Cristina Satriano, Placido Mineo

**Affiliations:** 1 Polymer Laboratory, Department of Chemical Sciences, University of Catania, Viale A. Doria 6, I-95125 Catania, Italy; 2 NanoHybrid Biointerfaces Lab (NHBIL), Department of Chemical Sciences, University of Catania, Viale A. Doria 6, I-95125 Catania, Italy; 3 CNR-IPCF Istituto per i Processi Chimico-Fisici, Viale F. Stagno d’Alcontres 37, I-98158 Messina, Italy; 4 CNR-IPCB Istituto per i Polimeri, Compositi e Biomateriali, Via P. Gaifami 18, I-95126 Catania, Italy

**Keywords:** multifunctional polymer-conjugate, all-trans retinoic acid, ATRA, folic acid, rhodamine B, polymer conjugates, polyvinyl alcohol, neuroblastoma, SH-SY5Y

## Abstract

Polymer-based systems have been demonstrated in novel therapeutic and diagnostic (theranostic) treatments for cancer and other diseases. Polymers provide a useful scaffold to develop multifunctional nanosystems that combine various beneficial properties such as drug delivery, bioavailability, and photosensitivity. For example, to provide passive tumour targeting of small drug molecules, polymers have been used to modify and functionalise the surface of water-insoluble drugs. This approach also allows the reduction of adverse side effects, such as retinoids. However, multifunctional polymer conjugates containing several moieties with distinct features have not been investigated in depth. This report describes the development of a one-pot approach to produce a novel multifunctional polymer conjugate. As a proof of concept, we synthesised polyvinyl alcohol (PVA) covalently conjugated with rhodamine B (a tracking agent), folic acid (a targeting agent), and all-trans retinoic acid (ATRA, a drug). The obtained polymer (PVA@RhodFR) was characterised by MALDI-TOF mass spectrometry, gel permeation chromatography, thermal analysis, dynamic light-scattering, NMR, UV-Vis, and fluorescence spectroscopy. Finally, to evaluate the efficiency of the multifunctional polymer conjugate, cellular differentiation treatments were performed on the neuroblastoma SH-SY5Y cell line. In comparison with standard ATRA-based conditions used to promote cell differentiation, the results revealed the high capability of the new PVA@RhodFR to induce neuroblastoma cells differentiation, even with a short incubation time and low ATRA concentration.

## 1. Introduction

In the last few decades, multifunctional nanosystems have emerged as a new paradigm in nanomedicine to confront the complexity of various diseases and treatments, including the inherent toxicity of some drugs, by the integration of multimodal imaging and therapeutic functions within a single therapeutic and diagnostic (theranostic) platform [1,2,3]. Polymer-based nanotechnologies have been demonstrated in the theranostic field [4,5], not only against cancer [6] but also for other diseases such as AIDS/HIV and multiple sclerosis [7,8]. The polymer-based systems used in nanomedicine offer a high versatility due to their multi-functionalities [7] and allow the combination of features from organic and inorganic components [4,9]. This makes them ideal for several applications such as improving drug bioavailability [10], integrating image contrast [11], and photosensitiser agents [12,13], as well as targeting moieties for the specific delivery at the site of action [14]. Conjugation with polymers can be exploited to trigger the biodistribution, elimination, and rate of metabolism of covalently bound drugs. Accordingly, soluble polymer conjugates have been introduced into clinical practice for several years now [15]. These water-soluble hybrid constructs, designed for intravenous administration, mostly fall into the two main categories of polymer–protein conjugates and polymer–drug conjugates and have demonstrated promising potential in nanomedicines for the diagnosis and treatment of cancer [16]. Furthermore, polymer conjugation to proteins reduces immunogenicity, prolongs plasma half-life, and enhances protein stability. On the other hand, polymer–drug conjugation promotes passive tumour targeting through the enhanced permeability and retention (EPR) effect and, at the cellular level, endocytic capture and lysosomotropic drug delivery [17]. To develop a polymer conjugate, it is necessary to choose the most suitable polymer backbone, the drug needed for the specific illness, and the targeting agent that leads the drug to the specific site.

Poly(lactic-co-glycolic acid) (PLGA), polyethylene glycol (PEG), polyvinyl alcohol (PVA), poly-l-lactic acid (PLA), polycaprolactone (PCL), and chitosan are the most common polymers used in drug delivery due to their biocompatibility, biodegradability, and the ease of functionalisation [18,19,20,21,22]. Among these, PVA is probably the most well-known biocompatible synthetic polymer, which has been used in the medical and biomedical fields, in bioartificial organs and tissues, contact lenses, and artificial cartilage [23,24,25,26,27]. Moreover, it has been commonly used as a carrier in drug delivery [28,29,30,31] and has already been approved for several clinical trials [19]. From a chemical point of view, the presence of numerous hydroxyl side groups in the PVA backbone leads to the ease of functionalisation with several organic and inorganic moieties [32,33].

Folic acid (FA) is commonly used as a targeting agent to treat solid tumours [34,35,36] because the folate receptor is over-expressed in human neoplasia tissues such as breast, renal, colon, pulmonary, and brain tumours. The interaction of the targeting agents with specific receptors over-expressed in diseased cells leads to the polymer conjugate endocytic internalisation and bioaccumulation. As an example, folic acid-modified electrospun PVA/polyethyleneimine (PEI) nanofibers have been demonstrated for cancer cell capture applications [37].

Imaging techniques are another fundamental aspect of biomedical treatments because they enable the visualisation and localisation of diseased tissues and allow physicians to follow the progress during treatment. Various fluorescent probes are commonly used for imaging tumours, including tetraphenylethylene [38], porphyrin [13,39,40], fluorescein [41], and rhodamine B [42]. 

Retinoids are an important class of molecules in the field of cancer treatment and are used in differentiation therapy [43]. In particular, the all-trans retinoic acid (ATRA) is used in several carcinoma types due to the ability to induce cellular differentiation and apoptosis by activating specific genomic pathways or influencing key signalling proteins [44], inducing growth arrest and differentiation of neuroblastoma (SH-SY5Y cell line, associated with the most common solid tumours in children) to neuron-like cells [45]. Therefore, ATRA is one of the most widely employed treatments for high-risk neuroblastoma patients, for targeting this population of cells and diminishing the growth potential of the brain cancer [46,47]. However, other than drug resistance mechanisms, some ATRA properties may limit its clinical efficacy, such as its hydrophobic nature, which does not allow parenteral administration, its propensity to degrade, owing to the susceptibility to light, heat, and oxidants, and the short biological half-life in humans (t_1/2_ = 45 min) caused by its metabolism regulation in the liver [48]. Hence, various targeted drug delivery platforms have been formulated to overcome the disadvantages of ATRA treatment, with nanoformulation that encompasses liposomes, solid lipid nanoparticles, polymer nanoparticles and lipid-coated inorganic nanoparticles [48]. Nevertheless, the non-covalent formulation of ATRA could result in an uncontrolled release of the drug and a sudden increase in concentration and availability, producing adverse side effects [28,49,50]. Covalent ATRA polymer conjugates may overcome this issue [28,51,52,53].

**Figure 1:**
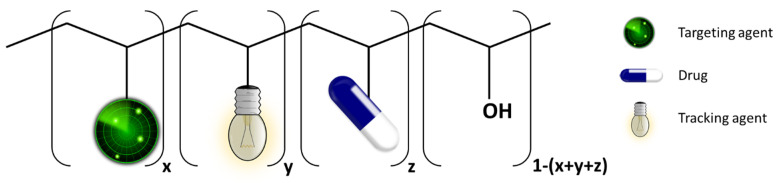
Sketch of the proposed multifunctional polymer conjugate structure. The terms x, y, and z indicate the molar fraction in the statistical polymer.

In the current work, a simple one-pot reaction was developed to produce a novel multifunctional polymer conjugate that is suitable as a carrier, tracker, and targeting agent (see Figure 1), able to carry the drug to a specific site. Specifically, free radical polymerisation was used to obtain PVAc and, by hydrolysis, PVA. Moreover, as a proof of concept, the hydroxyl side groups were exploited to covalently link the active moieties through a one-pot reaction, producing a multifunctional theranostic system containing ATRA as a differentiating agent, rhodamine B as a tracking agent, and FA as a targeting agent.

The obtained polymeric conjugate (namely PVA@RhodFR) and its precursors were characterised by matrix-assisted laser desorption/ionisation—time of flight (MALDI-TOF) mass spectrometry, gel permeation chromatography (GPC), thermal analyses, dynamic light scattering (DLS) measurements, nuclear magnetic resonance (NMR), UV-visible (UV-Vis), and fluorescence spectroscopy. To evaluate the bioactivity of the polymeric conjugate, cellular differentiation treatments were performed on the neuroblastoma SH-SY5Y cell line and verified by optical microscopy. These experiments allowed us to assess the neurite outgrowth of the samples over time and scrutinise the neurite morphology by confocal laser microscopy using a fluorescent structural marker, namely, actin green, which has a high affinity for the cytoskeletal actin protein. Compared to the standard condition that is already used to promote the formation of neuron-like cells [54], the biological response to PVA@RhodFR revealed the ability of the new polymeric conjugate to induce neuroblastoma cells differentiation, even for a short incubation time and low ATRA concentration, highlighting the promising potential of this new compound in brain cancer treatment. In addition to developing ATRA–polymer conjugates, the proposed approach was also exploited to obtain water-soluble conjugates that are useful in theranostics by covalently linking different on-demand drugs and targeting and tracking agents to the PVA chain.

## 2. Material and Methods

### 2.1. Materials

All the solvents, vinyl acetate, Azobisisobutyronitrile (AIBN), sodium hydroxide, N,N′-Dicyclohexylcarbodiimide (DCC), N,N-Dimethylpyridin-4-amine (DMAP), Rhodamine B, Folic acid, All-trans Retinoic acid (ATRA), were purchased from Sigma-Aldrich (Merck Group, Milan, Italy).

For the cellular experiments, Dulbecco’s modified eagle medium (DMEM)-F12, DMEM high glucose, penicillin-streptomycin solution, l-glutamine, foetal bovine serum (FBS), Dulbecco’s phosphate-buffered saline (PBS), bovine serum albumin (BSA), and dimethyl sulfoxide (DMSO) were purchased from Sigma-Aldrich (Merck Group, Milan, Italy). 3-(4,5-dimethyl-2-thiazolyl)-2,5-diphenyl-2H-tetrazolium bromide (MTT reagent) was obtained from AppliChem ITW Reagents (Nova Chimica Srl, Milan, Italy).

### 2.2. Instrumentation

MALDI-TOF mass spectra were acquired with a Voyager DE (PerSeptive Biosystem, Perkin Elmer, Waltham, MA, USA) equipped with the delay extraction device [55,56] (25 kV applied after 2600 ns, potential gradient 454 V mm^−1^, wire voltage 25 V), detecting ions in linear mode. The mass spectrometer calibration procedure was previously reported [57]. Weight average molecular weights were determined using a Grams/386 software (Version 3.04, Galactic Industries Corp, Salem, NH, USA) applied on the spectra, following a previously reported method [58], corrected for baseline and the offset. Trans-2-[3-(4-tert-Butylphenyl)-2-methyl-2-propenylidene]malononitrile (DCTB) was used as matrix.

Proton nuclear magnetic resonance (^1^H-NMR) and proton–proton homonuclear correlation spectroscopy (COSY) spectra were obtained using a ^UNITY^INOVA Varian instrument operating at 500 MHz (^1^H), using VNMR 6.1C software for acquisition and spectra processing. The samples were dissolved in deuterated chloroform (CDCl_3_) or deuterated water (D_2_O), and the chemical shifts were expressed in ppm and compared with the chloroform (CHCl_3_) or water (H_2_O) residue signal. 

Average molecular weights and molecular weight distributions were determined by Gel Permeation Chromatography (GPC), with a PL-GPC 110 (Polymer Laboratories, Agilent Technologies, Santa Clara, CA, USA) thermostated at 35.0 ± 0.1 °C, equipped with two columns joined in series (mixed-D and one mixed-E PL-gel 5 mm) and a set of three detectors composed of a UV-visible spectrophotometer (Hewlett Packard series 1050), a DAWN multiangle laser light scattering (Wyatt Technology, Santa Barbara, CA, USA), and a refractive index (Polymer Laboratories, Agilent Technologies, Santa Clara, CA, USA). The samples were analysed using tetrahydrofuran (THF) as eluent (1 mL min^–1^). The data were analysed employing the software ASTRA 6.0.1.10 (Wyatt Technology, Santa Barbara, CA, USA), using a *dn*/*dc* value of 0.054 mL g^–1^ for poly vinyl-acetate (PVAc) in THF [59,60]. 

UV-Visible spectra were acquired using a Cary60 UV-vis spectrophotometer (Agilent Technologies, Santa Clara, CA, USA), and the fluorescence spectra were recorded through an FP-8200 spectrofluorimeter (Jasco Corporation, Tokyo, Japan), at 25 ± 0.1 °C, using quartz cuvettes with a 1 cm path length and water (LC-MS grade) or Dulbecco’s phosphate-buffered saline (PBS) as solvents. The spectra were processed using the Spectragryph optical spectroscopy software [61].

Thermogravimetric measurements were performed with a Perkin-Elmer TGA 7 equipped with a TAC 7/DX (Perkin Elmer, Waltham, MA, USA), applying a thermal ramp of 10 °C min^−1^ (range 50–800 °C) in a nitrogen atmosphere (60 mL min^−1^).

Differential scanning calorimetry (DSC) measurements were performed with a TA Q20 equipped with a Refrigerant Cooling System (TA instruments, New Castle, DE, USA), applying a heating rate of 10 °C min^−1^, in an anhydrous nitrogen atmosphere (60 mL min^−1^).

Dynamic Light Scattering (DLS) analyses were performed with a miniDAWN Treos (Wyatt Technology, Santa Barbara, CA, USA) multiangle laser light scattering detector, equipped with a Wyatt QELS DLS Module, at 25 °C using water as solvent. Particle size distributions were analysed through the software ASTRA 6.0.1.10 (Wyatt Technology, Santa Barbara, CA, USA).

### 2.3. Synthesis of the Poly Vinyl-Acetate 

The Polyvinyl acetate (PVAc) was synthetised through a bulk radical polymerisation. Briefly, a suitable amount of vinyl acetate monomer was previously purified to eliminate the inhibitor and the adsorbed water using a chromatographic column filled with a double layer of anhydrous sodium sulphate and basic alumina. The freshly purified vinyl acetate (9.56 g, 111 mmol) was added with Azobisisobutyronitrile (AIBN, 202.55 mg, 1.233 mmol) as a thermal initiator into a flask in Argon atmosphere. Then, the mixture was heated under stirring into an oil bath at 60 °C for four hours. The colourless solid obtained was solubilised in THF (20 mL) and precipitated in cold hexane to separate the polymer fraction. The residue was dried under vacuum at 50 °C.

^1^H-NMR analysis (500 MHz, 27 °C, CDCl_3_): 5.05–4.76 ppm (1 H, CH, b), 2.13–1.94 ppm (3 H, CH_3_, c), 1.93–1.62 ppm (2 H, CH_2_, a). The spectrum is reported in Figure S1. 

GPC analysis: Mn = 7.58 × 10^4^ Da, Mw = 1.10 × 10^5^ Da, and PDI = 1.46. Thermogravimetric analysis: T_onset_ = 317 °C; PDT = 337.7 °C; residue at 600 °C = 5%. DSC: Tg = 43.0 °C.

### 2.4. Synthesis of Copoly-Vinyl Acetate-Vinyl Alcohol

The copoly-vinyl acetate-vinyl alcohol (CoVAc-VA) was obtained through alkali-catalysed saponification of the PVAc in methanol. Briefly, the PVAc (1.1689 g, 13.58 mmol) was dissolved in methanol (8 mL); then, 390 μL of a methanolic solution of NaOH (0.29 mmol) was added. The reaction was performed for five minutes at room temperature. Then, it was precipitated and then copiously washed using diethyl ether, and finally dried under vacuum, at 50 °C for 24 h. 

^1^H-NMR analysis (Figure S3, 500 MHz, 27 °C, CDCl_3_): 5.24–4.78 ppm (1 H, C-H, b,d), 2.18–1.95 ppm (2.71 H, CH_3_, e), 1.93–1.62 ppm (2.82 H, CH_2_, a,c). Notably, the a and c signals are overlapped with the imbibed water residual signal (1.65 ppm). From the integration of the NMR signals, the hydrolysis grade was calculated, resulting in about 10% in repetitive units. 

GPC analysis: Mn = 9.42 × 10^4^ Da, Mw = 1.30 × 10^5^ Da, and PDI = 1.38. Thermogravimetric analysis: T_onset_ = 314 °C; PDT = 342 °C; residue at 600 °C = 5%. DSC: Tg = 48.7 °C. 

### 2.5. Synthesis of Polyvinyl Alcohol

The polyvinyl-alcohol (PVA) was obtained similarly to the CoVAc-VA, using a PVAc/NaOH weight ratio of 4:1 in methanol. The PVAc (2.55 g, 29.62 mmol) was solubilised in methanol (17 mL); then, the NaOH (0.64 g, 15.94 mmol) was dissolved in methanol (5 mL) and added to the polymer solution. The reaction was conducted for 15 min at room temperature. The white solid obtained was washed copiously with methanol and dried under vacuum, at 50 °C. The characterisation of the PVA ensures the total hydrolysis of the PVAc, as confirmed by NMR and FT-IR analysis. 

^1^H-NMR analysis (Figure S4, 500 MHz, 27 °C, D_2_O): 4.01–3.35 ppm (1 H, C-H, b); 3.21 ppm (methanol residue); 1.78 ppm (0.03 H, acetate residual, O-CH_3_); 1.72–1.22 ppm (2 H, CH_2_, a).

Thermogravimetric analysis: T_onset_ = 214 °C; PDT = 240 °C; residue at 600 °C = 13%. 

### 2.6. Synthesis of the Copolymer CoVAc-VA Functionalised with Rhodamine B 

The functionalisation of the CoVAc-VA was performed by Steglich esterification. Briefly, the CoVAc-VA (100 mg) was solubilised in 1.22 mL of anhydrous dichloromethane. Dry solvents are needed to avoid the degradation of the coupling agent N,N′-Dicyclohexylcarbodiimide (DCC). Then, the mixture was stirred in an ice bath for five minutes. N,N-Dimethylpyridin-4-amine (DMAP, 0.25 mg, 2 μmol) and Rhodamine B (19.49 mg, 40.7 μmol) were added and stirred at 0 °C for five minutes. Then, DCC (8.4 mg, 40.7 μmol) was added, and the reaction was performed at room temperature (25 °C) for seven hours.

The mixture was dried under N_2_, then solubilised in THF and precipitated in water. The solid obtained was washed copiously with water until no Rhodamine B was present in the UV-vis analysis of the supernatant. The obtained pink solid powder was dried under vacuum, at 50 °C. Then, the obtained CoVAc-VA@Rhod was characterised through GPC analysis. More details are reported in Section 3.

### 2.7. One-Pot Synthesis of the Water-Soluble Polymer Conjugates

The PVA was used to produce two polymer conjugates by Steglich esterification: the first functionalised with Rhodamine B (PVA@Rhod), the second with Rhodamine B, Folic acid, and ATRA (PVA@RhodFR).

PVA@Rhod synthesis: The PVA (0.1 g, 2.27 mmol in VA) was solubilised in 2 mL of anhydrous Dimethyl sulfoxide (DMSO). Then, in a nitrogen atmosphere, Rhodamine B (32 mg, 68 μmol) and DMAP (1 mg, 10 μmol) were added. After five minutes of stirring, DCC (14 mg, 68 μmol) was added. The reaction was maintained under stirring for three days at room temperature and then was stopped by precipitation in THF (12 mL). The solid obtained was washed with acetone under sonication and separated by centrifugation until the supernatant showed no trace of the reactant (verified by UV-vis spectroscopy).

PVA@RhodFR synthesis: The PVA (0.1 g, 2.27 mmol) was solubilised in 2 mL of anhydrous DMSO. Then, in nitrogen atmosphere, Rhodamine B (32.66 mg, 68 μmol), Folic acid (15.05 mg, 34 μmol), ATRA (20.48 mg, 68 μmol), and DMAP (1.04 mg, 8.5 μmol) were added. After five minutes of stirring at room temperature, DCC (35.16 mg, 0.17 mmol) was added. The reaction was maintained under stirring for three days at room temperature, then was stopped by precipitation in ethanol. The solid obtained was washed with ethanol under sonication and separated by centrifugation until the supernatant showed no trace of the reactants (verified by UV-vis spectroscopy).

Both PVA@Rhod and PVA@RhodFR were characterised using UV-vis, fluorescence, and ^1^H-NMR spectroscopies (see Section 3).

### 2.8. Cell Cultures

Human neuroblastoma (SH-SY5Y) cells were cultured in Dulbecco’s modified eagle medium (DMEM)-F12 medium supplemented with 10% (*v*/*v*) Fetal Bovine Serum (FBS), 2 mM L-glutamine, and 100 U penicillin/0.1 mg/mL streptomycin. Cells were grown in tissue-culture-treated Corning^®^ flasks (Merck Group, Milan, Italy) under a humidified atmosphere of air/CO_2_ (95:5) at 37 °C in an incubator (Heraeus Hera Cell 150C incubator, Thermo Fisher Scientific, Waltham, MA, USA). 

### 2.9. Confocal Microscopy Analysis and Differentiation of SH-SY5H Cells

SH-SY5Y cells were seeded on glass-bottom dishes (WillCo-dish^®^, Willco Wells B.V., Amsterdam, The Netherlands) with 22 mm of glass diameter, at a density of 4.8 × 10^4^ cells per dish, in DMEM-F12 medium with 10% (*v*/*v*) FBS. After 24 h of plating, the medium was changed with DMEM high glucose supplemented with 0.5% (*v*/*v*) FBS and cells were treated for 5 h and 72 h, respectively, with PVA@RhodFR sample at the concentrations of 2.9 × 10^−7^ M and 1.16 × 10^−9^ M for the polymer and retinoic acid, respectively. 

As positive controls, we used ATRA at the two concentrations of 1.16 × 10^−9^ M (i.e., comparable to that conjugated to the polymer) as well as of 1 × 10^−5^ M, which is the standard condition to promote neuroblastoma cells differentiation [54,62,63]. Additionally, control samples of PVA@Rhod (2.4 × 10^−7^ M) and PVA@Rhod +RA (i.e., the physical mixture of the polymer and ATRA, at the final PVA@Rhod concentration of 2.4 × 10^−7^ M, while ATRA was added at the two concentrations of 1.16 × 10^−9^ M and 1 × 10^−5^ M, respectively) were used. Optical bright field images were recorded with a Leica ICC50 W microscope immediately after the treatment (t = 0) and after 5 h and 72 h of incubation and analysed, to assess the neurite outgrowth, using the NeuronJ (neurite tracing and quantification) plugin from the ImageJ 1.8.0 Software (NIH, Bethesda, MD, USA). Following the incubation time, cells were fixed with high purity 2% (*w*/*v*) paraformaldehyde in Dulbecco’s phosphate-buffered saline (PBS) and then stained with nuclear dye Hoechst33342 (1 µg/mL). 

Afterwards, cells were permeabilised with 0.5% (*v*/*v*) Triton X-100 supplemented with 0.1% (*w*/*v*) BSA in PBS and then stained with a high-affinity F-actin probe, conjugated to green-fluorescent Alexa Fluor 488 dye (2 drops/mL) (Thermo Fisher Scientific, Waltham, MA, USA).

Confocal imaging microscopy was performed with an Olympus FV1000 confocal laser scanning microscope (LSM), furnished with diode UV (405 nm, 50 mW), multiline Argon (457 nm, 488 nm, 515 nm, total 30 mW), HeNe(G) (543 nm, 1 mW), and HeNe(R) (633 nm, 1 mW) lasers. An oil immersion objective (60xO PLAPO) and spectral filtering systems were used. The detector gain was fixed at a constant value and images were collected, in sequential mode, randomly all through the area of the dish. All images were deconvolved with Huygens Essential 4.0 software (Scientific Volume Imaging, Hilversum, The Netherlands).

### 2.10. Cell Viability Assay

To perform the cell viability assay (MTT), cells were seeded at a density of 1.5 × 10^4^ cells/well into a 96-well plate and maintained in (DMEM)-F12 medium supplemented with 10% (*v*/*v*) FBS. The day after, cells were treated for 24 h and 48 h in (DMEM)-F12 with 1% (*v*/*v*) FBS with PVA@RhodFR (polymer/ATRA concentrations of 1.4 × 10^−7^ M/2.3 × 10^−9^ M, 2.9 × 10^−7^ M/1.2 × 10^−9^ M, 5.8 × 10^−7^ M/5.8 × 10^−10^ M). As positive controls, cells were treated with ATRA (at the concentrations of 2.3 × 10^−9^ M, 1.2 × 10^−9^ M, and 5.8 × 10^−10^ M) and the mixture of PVA@Rhod + ATRA (at the polymer/ATRA concentrations of 1.2 × 10^−7^ M/5.8 × 10^−10^ M, 2.4 × 10^−7^ M/1.2 × 10^−9^ M, and 4.8 × 10^−7^ M/2.3 × 10^−9^ M, respectively).

After 24 h and 48 h of incubation, cells were washed with buffer and treated with 5 mg/mL of 3-(4,5-dimethyl-2-thiazolyl)-2,5-diphenyl-2H-tetrazolium bromide at 37 °C for 90 min. Afterwards, the formazan salts formed by succinate dehydrogenase activity in live cells were solubilised with DMSO and quantified spectrophotometrically by a Synergy 2 microplate reader (BioTek, Winooski, VT, USA), by the absorbance value at a wavelength of 570 nm. All conditions were measured in triplicate and results were expressed as % of viable cells over the negative control (i.e., untreated cells).

## 3. Results and Discussion

### 3.1. Synthesis and Characterisation of the Polymer Conjugate

**Figure 2:**
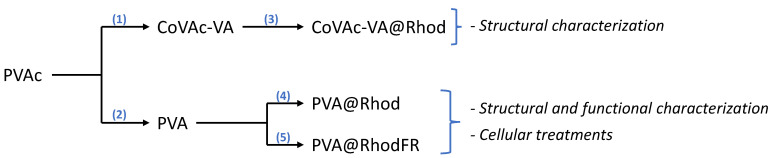
Representative scheme of the conjugated polymer synthesis routes: the products (1) and (2) were obtained by alkali-catalysed saponification; then, products (3), (4), and (5) were obtained by functionalisation of the related polymer backbones by catalysed esterification.

The multifunctional conjugated polymers were obtained following the routes shown in Figure 2, starting from the synthesis of PVAc by bulk radical polymerisation using AIBN as a thermal initiator (see the Section 2.3 for details). This approach was chosen due to the reliability of the synthetic protocol and the resulting polymer structure. The polymeric nature of the PVAc was ascertained by GPC, showing a number average molecular weight of Mn = 7.58 × 10^4^ Da (polydispersity index 1.46). 

The PVAc structure was determined by MALDI-TOF analysis (Figure S2). The spectrum of PVAc shows a series of peaks at *m*/*z* 693 + n86 (detected as M-Na^+^, *, *n* = 7–31) and at *m*/*z* 709 + n86 (detected as M-K^+^, #, *n* = 7–27), which are related to oligomeric chains with isobutyronitrile and saturated or unsaturated end groups, as expected due to the radical disproportionation reactions. The PVAc structure was also confirmed by ^1^H-NMR characterisation (see Section 2.3 and Figure S1). 

**Figure 3:**
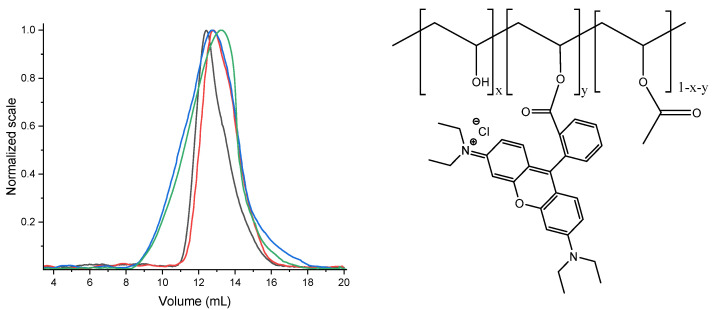
(**Left**) GPC RI detector traces of PVAc (black line), CoVAc-VA (red line), and CoVAc-VA@Rhod (blue line); GPC UV-vis detector trace (set at 554 nm, maximum absorption band of rhodamine B, green line) of CoVAc-VA@Rhod. The CoVAc-VA did not show UV-vis absorption at 550 nm. (**Right**) molecular structure of the CoVAc-VA@Rhod copolymer.

Subsequently, two derivatives were produced from PVAc: a partially hydrolysed VAc-VA copolymer (CoVAc-VA) and a fully hydrolysed polymer (PVA). The CoVAc-VA was used as a model to verify the efficiency of the reaction for conjugation, whereas the PVA was used as the polymeric backbone for the multifunctional conjugation. The partial hydrolysis of PVAc was performed in methanol (by alkali-catalysed saponification, Figure 2, step 1) obtaining a CoVAc-VA suitable for linking a side-chain molecular system. The molecular mass distribution of the copolymer was determined by GPC analysis (Figure 3), exhibiting a number average molar mass of Mn = 9.42 × 10^4^ Da (PDI = 1.38), which is slightly lower than that of the parent PVAc due to its partial hydrolysis and the consequent loss of acetate moieties. The copolymer was also characterised by ^1^H-NMR analysis (see Section 2.4 and Figure S3), showing a hydrolysis grade of about 10% in repetitive units.

To bind the rhodamine B (used as a tracking agent) to CoVAc-VA (Figure 2, step 3), the Steglich reaction was conducted in anhydrous dichloromethane using DCC and DMAP as catalysts, obtaining the CoVAc-VA@Rhod system. GPC analysis (Figure 3) of the CoVAc-VA@Rhod was performed using a triple-detector configuration (refractive index (RI), MALLS, and UV-vis) to verify the presence of rhodamine B as a side-chain moiety. The refractive index detector (Figure 3, blue line) shows a broad band from 8 to 18 mL. The signal of the UV-Vis detector (λ at 554 nm—Figure 3, green line), connected in parallel to the RI, quasi-overlapped with the RI trace, confirming the homogeneous distribution of rhodamine B into the copolymer chain. 

**Figure 4:**
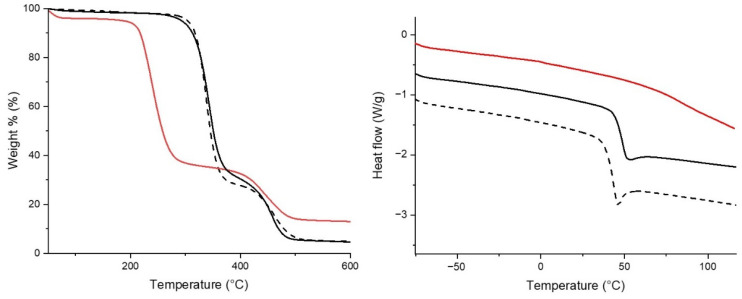
TGA (**left**) and DSC traces (**right**) of PVAc (black dashed line), CoVAc-VA (black continuous line), and PVA (red continuous line).

To synthesise the multifunctional polymer conjugate, a fully hydrolysed PVAc was performed in NaOH methanolic solution. The ^1^H-NMR analysis of PVA (see Section 2.5 and Figure S4) confirmed the occurrence of almost total hydrolysis (>99%). As expected, due to the different side groups, the hydrolysis process and degree of hydrolysis influence the thermal behaviour of the polymer. The thermogravimetric analysis of CoVAc-VA (Figure 4 left, continuous black line) shows the main loss at about 340 °C and a minor one at about 460 °C, with a behaviour similar to PVAc (Figure 4 left, dashed black line). The first weight loss is mainly due to the thermal deacetylation [64]. The second decomposition step, at about 445 °C, is due to the breakdown of the residual polymer backbone. In contrast, the PVA trace (Figure 4 left, red line) shows an initial weight loss (below 100 °C) due to the water loss, and the main weight loss at about 240 °C is due to the de-hydration of the PVA backbone [64], followed by a minor loss at about 445 °C. 

The thermal behaviour differences among the polymers are also evident from DSC analyses. DSC traces (Figure 4 right) of the copolymer show a slightly higher glass transition temperature (Tg) compared with the PVAc (48.7 and 43.7 °C, respectively), while PVA samples do not show a clear glass transition.

As a proof of concept, the multifunctional polymer conjugate (PVA@RhodFR) was designed with rhodamine B as a tracking agent, folic acid as a targeting agent, and all-trans retinoic acid (ATRA) as an active drug. The polymeric conjugate was synthesised using a simple one-pot reaction, employing DCC and DMAP as catalysts (see Section 2.6). To obtain a system that was useful as a blank in cellular experiments, a simpler polymer conjugate was also produced by linking rhodamine B to the PVA backbone through the same one-pot synthesis approach (PVA@Rhod). 

**Figure 5:**
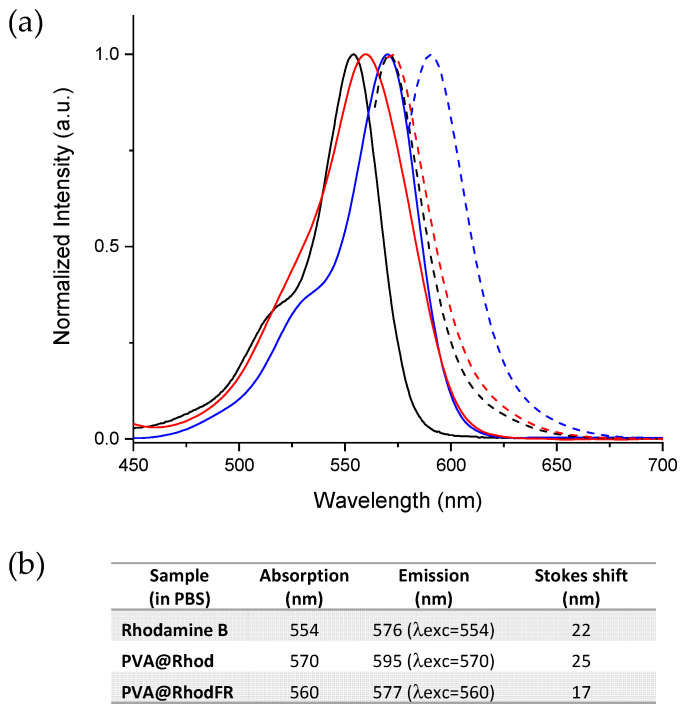
(**a**) Solid lines, normalised UV-Vis spectra for water solutions of rhodamine B (black), PVA@Rhod (blue), and PVA@RhodFR (red). Dashed lines, normalised fluorescence spectra for water solutions of rhodamine B (black, λ_exc_ = 554 nm), PVA@Rhod (blue, λ_exc_ = 570 nm), and PVA@RhodFR (red, λ_exc_ = 560 nm). (**b**) The absorption and emission wavelengths of the samples, and the related Stokes shift.

The optical properties of the dye and polymer conjugates were studied by UV-Vis and fluorescence spectroscopy, using water as a solvent. The rhodamine B shows the typical absorption of the xanthene moiety, with a peak at 554 nm and a shoulder at 574 nm, both attributed to the S0-S1 transition [65] (Figure 5a, black line). The molar extinction coefficient of rhodamine B in water was determined using the Lambert–Beer law (109,000 M^−1^cm^−1^). The absorption band of the rhodamine B covalently linked to the polymer (PVA@Rhod, Figure 5a, blue line) is slightly redshifted (absorption maximum at 570 nm) with respect to the free rhodamine (554 nm). However, the rhodamine B linked in PVA@RhodFR (Figure 5a, red line) shows the maximum absorption at 560 nm. The observed redshift with respect to the absorption of free rhodamine is due to the structural and electronic modification of the ester group [65]. The mol% content of the rhodamine B within the polymer conjugates was calculated by UV-Vis using the rhodamine B molar extinction coefficient, resulting in about 0.6% (mol) for both the PVA@Rhod and the PVA@RhodFR.

Even the fluorescence emission (dashed line in Figure 3) of the PVA@Rhod (595 nm, λ_exc_ = 570 nm) and the PVA@RhodFR (577 nm, λ_exc_ = 560 nm) are redshifted and show different Stokes shifts compared with free rhodamine B (576 nm, λ_exc_ = 554 nm). The differences in the Stokes shifts of the polymer conjugates (see Figure 5b) are a further confirmation of the covalent bond between the dye and the polymer, and they are proof of the chemical–physical interaction between the moieties. It is already known that the Stokes shift is related to the relaxation of the molecular excited state and the electronic-vibrational modes. This means that a higher molecular rigidity leads to smaller Stokes shifts [66]. The experimental data states that PVA@Rhod and PVA@RhodFR maintain the optical properties of the chromophore. The optical properties of the polymer conjugates were also verified in PBS Dulbecco medium (used in cellular treatments to avoid osmosis phenomena), showing negligible variations compared with that exhibited in water solution.

**Figure 6:**
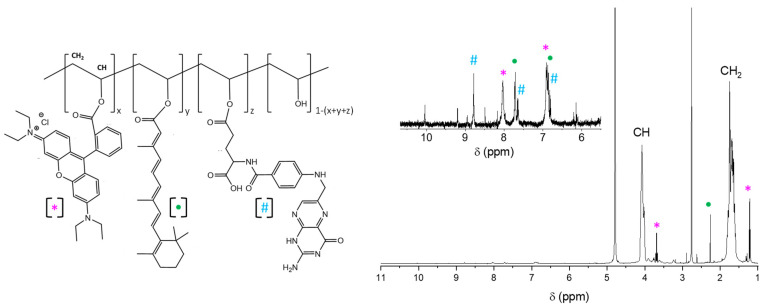
^1^H-NMR spectrum (**right**) of PVA@RhodFR (D_2_O)**,** with the respective molecular structure (**left**) for signals attributions (*, Rhodamine B; •, ATRA; #, Folic acid).

The polymer conjugates’ structures were investigated by ^1^H-NMR. The PVA@Rhod showed signals corresponding with the polymer backbone (4.30–3.45 ppm, 1 H, CH; 1.90–1.50 ppm, 2 H, CH_2_) and with the rhodamine B protons [67] (8.3–6.5 ppm). As expected, the PVA@RhodFR showed a more complex ^1^H-NMR spectrum (Figure 6). In addition to the well-known polymer backbone signals (4.30–3.45 ppm, 1 H, CH; 1.90–1.50 ppm, 2 H, CH_2_), several signals are recognisable due to the side-chain moieties rhodamine B [67] (marked with *), ATRA [68] (marked with •), and FA [69] (marked with #): (8.78 ppm, #; 8.03 ppm, *; 7.72 ppm, •; 7.64 ppm, #; 7.00–6.75 ppm, *, •, #; 3.69 ppm, *; 2.26 ppm, •; 1.22 ppm, *). The calculated degree of functionalisation with respect to the polymer’s repetitive units is: 0.60 mol% for rhodamine B, 0.40 mol% for ATRA and 0.15 mol% for FA. 

**Figure 7:**
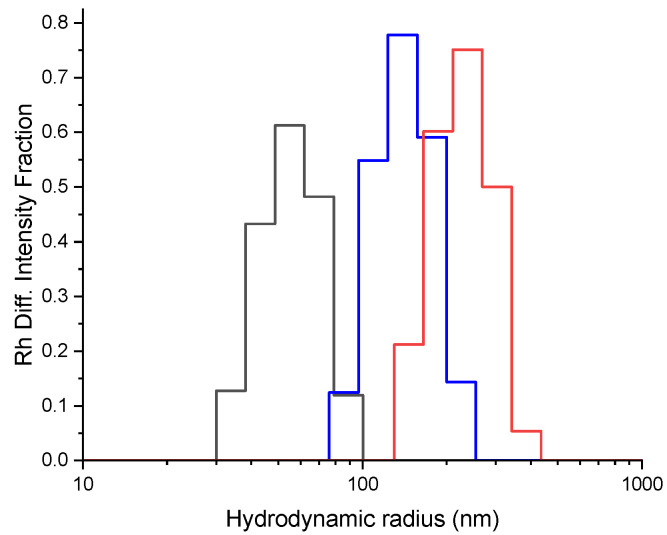
DLS measurements on PVA (black line), PVA@Rhod (blue line), and PVA@RhodFR (red line) aqueous solutions (0.1 g/L).

One of the most important characteristics of the polymer conjugates for theranostic applications is their ability to accumulate into diseased human tissues by means of the active or passive (exploiting the EPR effect) targeting properties [70]. To evaluate whether the size of the PVA and its derivatives meet the criteria to exploit the EPR effect, DLS measurements were performed. The PVA, due to the formation of the aggregates [71], exhibits a size distribution of the hydrodynamic radius centred at 54 nm (Figure 7, black line). Instead, DLS analysis conducted on polymer conjugates revealed higher sizes, with the PVA@Rhod (Figure 7, blue line) size distribution centred at 140 nm, and the PVA@RhodFR (Figure 7, red line) at 230 nm. Comparing the size distributions, the functionalisation of the polymer increases the size of the system in solution, probably because of the decrease in the number of hydrophilic groups in the PVA backbone. Notably, the size of PVA@RhodFR matches sufficiently with the necessary criteria to exhibit passive targeting [72,73,74]. Moreover, the Rg/Rh ratio value of 0.84 obtained for PVA@RhodFR suggests a sphere-like morphology.

### 3.2. Differentiation of SH-SY5Y Tumour Cells to Neuron-like Cells and Cytotoxicity Assay

**Figure 8:**
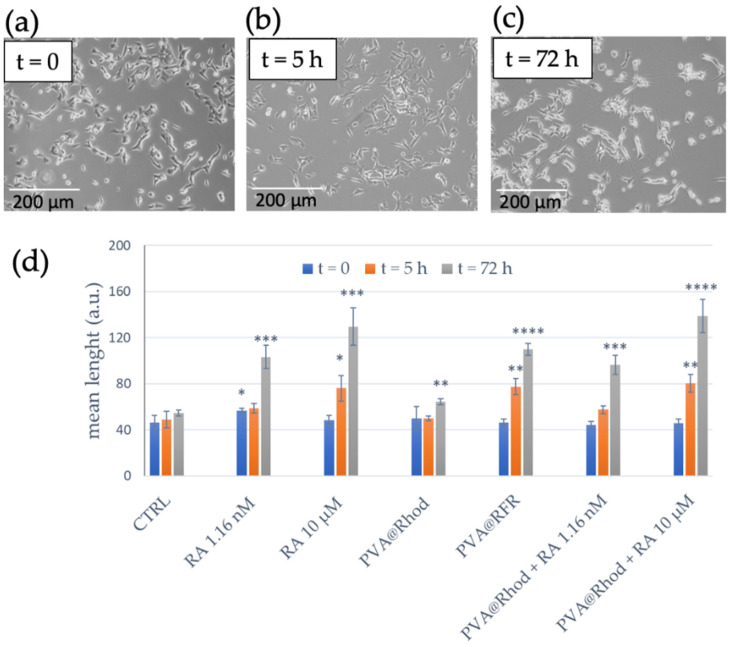
Representative optical phase-contrast images of the SH-SY5Y neuroblastoma cell line treated with PVA@RhodFR at the concentrations of 2.9 × 10^−7^ and 1.16 × 10^−9^ M for the polymer and retinoic acid, respectively, at t = 0 (**a**), t = 5 h (**b**), and after 72 h (**c**) of incubation (scale bar = 200 µm). Quantification of induced neurite outgrowth in response to the treatment of neuroblastoma cells (**d**) for 5 and 72 h with: 1.16 × 10^−9^ and 1 × 10^−5^ M ATRA; 2.4 × 10^−7^ M PVA@Rhod; PVA@RhodFR (2.9 × 10^−7^ M polymer; 1.16 × 10^−9^ M retinoic acid); PVA@Rhod + ATRA mixture (2.4 × 10^−7^ M polymer; 1.16 × 10^−9^ and 1 × 10^−5^ M ATRA). (*) *p* < 0.05, (**) *p* < 0.01, (***) *p* < 0.001, (****) *p* < 0.0001 vs. CTRL (Student’s *t*-test). Data represent mean ± SEM of at least 10 cells/experiment of *n* = 3 independent experiments.

To assess the ability of ATRA-functionalised PVA to traffic the active molecule within the cells, we tracked the differentiation process of neuroblastoma cells induced by the PVA@RhodFR for 72 h. Positive controls were included in the study, consisting of cells treated with ATRA alone and the simple mixture of PVA@Rhod and ATRA. Representative optical bright-field images were recorded immediately after the treatment (t = 0) and after 5 and 72 h of incubation, shown in Figure 8a,b. The outputs of the analyses to quantify the neurite outgrowth, obtained using the NeuronJ plugin of ImageJ Software, are given in Figure 8c. 

Notably, at the beginning of the experiment, SH-SY5Y cells display a neuroblast-like morphology with rounded and non-polarised cell bodies (Figure 8a). After 5 h of incubation with 10 µM ATRA, cells become morphologically more similar to primary neurons with long straight cell processes and varicosities [62,75,76]. The cell treatment with ATRA at the lowest concentration of 1.16 nM only leads to SH-SY5Y differentiation for the longest incubation time (t = 72 h), showing a lower branching rate and fewer connections of neurites in comparison to what was obtained with the 10 µM ATRA treatment. These findings are in agreement with the literature, demonstrating that SH-SY5Y cells treated with 10 µM ATRA differentiate into neuron-like cells within 48 h, due to the ATRA induction of several gene products, transcription factors, structural proteins, neurotransmitters, neuropeptide hormones, growth factors, enzymes, and cell surface receptors, thus displaying morphological changes and leading to the arrest of cell proliferation [77,78,79]. Remarkably, Figure 8c reveals that SH-SY5Y cells acquire neuron-like morphology with long neurites and branch outgrowth even after a short incubation time (t = 5 h) with PVA@RhodFR, similar to what was observed for treatments with the PVA@Rhod + ATRA mixture and the free ATRA (10 µM). 

The free ATRA molecules are present as aggregates in solution due to the compound’s low solubility in the aqueous medium. Such a low solubility decreases the aqueous concentration of ATRA, and thus, reduces the bioavailability and biological activity. The development of the PVA@RhodFR system can provide a continuous supply of well-dispersed active molecules to the cells due to its aqueous solubility, thereby explaining the higher bioavailability and biological efficacy of ATRA linked to PVA.

**Figure 9:**
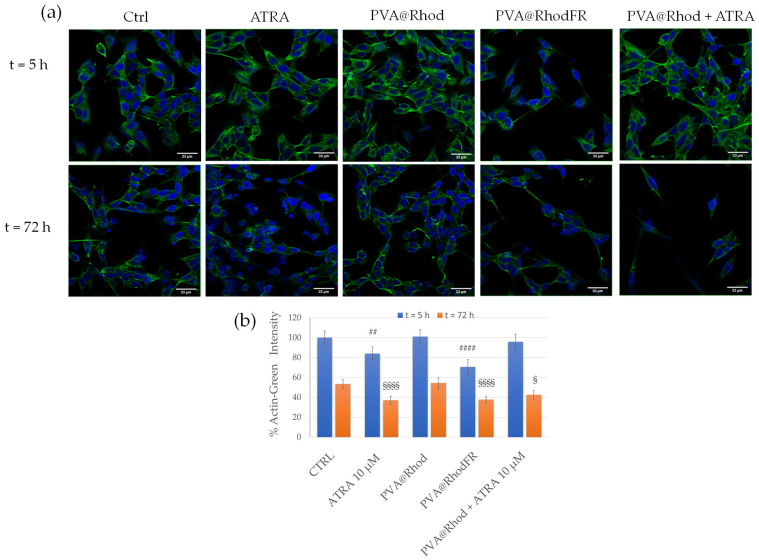
(**a**) LSM fluorescence micrographs (in blue, nuclear staining, λ_ex/em_ = 405/425–450 nm; in green, Actin Green, λ_ex/em_ = 488/500–530 nm) of neuroblastoma cells after 5 and 72 h of treatment with: 1 × 10^−5^ M ATRA; 2.4 × 10^−7^ M PVA@Rhod; PVA@RhodFR (2.9 × 10^−7^ M polymer; 1.16 × 10^−9^ M retinoic acid); PVA@Rhod + ATRA mixture (2.4 × 10^−7^ M polymer; 1.16 × 10^−9^ and 1 × 10^−5^ M ATRA). Scale bar = 33 μm. (**b**) Quantitative analysis of Actine-Green emission intensity. Data represent mean ± SEM of at least 15 cells/image for *n* = 3 different areas of the dish. Symbols represent the correlation significant at the (##) *p* < 0.01, (####) *p* < 0.0001 vs. CTRL 5 h; (§) *p* < 0.05, (§§§§) *p* < 0.0001 vs. CTRL 72 h (Student’s *t*-test).

To validate the hypothesis of SH-SY5Y differentiation induced by PVA@RhodFR, the neurite morphology was scrutinised by LSM using a fluorescent structural marker, namely, actin green, which has a high affinity for the cytoskeletal actin protein. As shown in Figure 9a, the cytoskeleton of untreated SH-SY5Y cells after 5 h displays a small area with short filaments of actin (F-actin), mostly confined at the cell membrane, and globular actin (G-actin). After 72 h, F-actin is less dominant and looks thinner, with an overall reduction in the emission intensity of the actin probe. The signal decrease is most likely due to a downregulation of the actin expression, leading to cytoskeleton remodelling, cell shrinkage, and loss of cell integrity, which are associated with the experimental conditions, namely, serum deprivation and long incubation time (0.5% *v*/*v* FBS for 72 h) (Figure 9a) [80]. Cells treated with 10 µM ATRA show the phenotype of differentiated cells with small, rounded cell bodies and thin, long filaments of actin, especially after 72 h of incubation, which leads to a significant reduction of the actin intensity compared with the negative control, i.e., untreated cells. 

The PVA@Rhod-treated cells exhibit no differences in terms of cytoskeleton reorganisation compared with untreated cells, whereas the 10 µM ATRA-treated cells are clearly differentiated, especially after 72 h incubation. Interestingly, the treatment with PVA@RhodFR at the ATRA concentration of 1.16 × 10^−9^ M, similar to the free 10 µM ATRA treatment, induces visible cytoskeletal actin modification, with an increased number and length of the structural protein filaments and a significant reduction of the actin intensity compared with the untreated cells, after both 5 h and 72 h of incubation. These data agree with the neurite quantification (Figure 9b).

**Figure 10:**
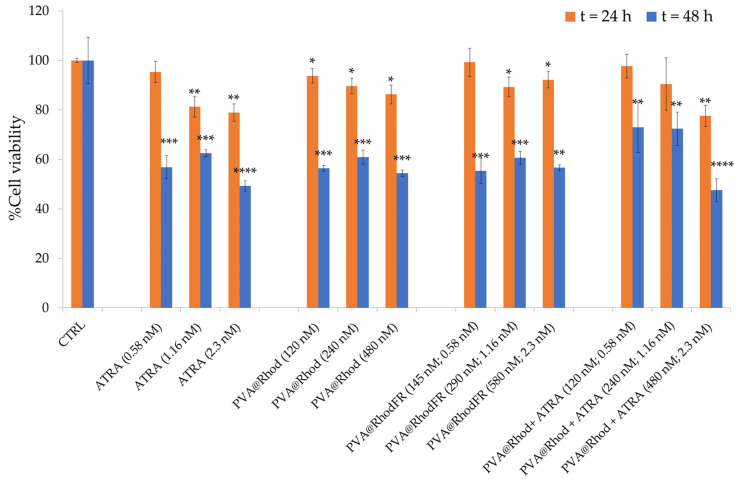
Cell viability assay (MTT) on SH-SY5Y cells untreated (negative control) or treated for 24 and 48 h with: ATRA (5.8 × 10^−10^ M, 1.16 × 10^−9^ M and 2.3 × 10^−9^ M); the fluorescent polymer PVA@Rhod (1.2 × 10^−7^ M, 2.4 × 10^−7^ M, 4.8 × 10^−7^ M); the functionalised polymer PVA@RhodFR (1.4 × 10^−7^ M, 2.9 × 10^−7^ M, 5.8 × 10^−7^ M); and the mixture PVA@Rhod + ATRA (1.2 × 10^−7^ M, 2.4 × 10^−7^ M, 4.8 × 10^−7^ M). The bars represent means ± S.D. of three independent experiments (S.D. = standard deviation). Statistical analysis was performed using the Student’s *t*-test. (*) *p* < 0.05, (**) *p* < 0.01, (***) *p* < 0.001, (****) *p* < 0.0001 vs. CTRL.

The cytotoxicity of PVA@RhodFR in SH-SY5Y was investigated by MTT assay and compared with the positive controls, consisting of the bare polymer (PVA@Rhod), ATRA alone, and the mixture (PVA@Rhod + ATRA). The results revealed a time-dependent and dose-dependent progressive loss of cell viability (Figure 10). After 24 h incubation with free ATRA, no cytotoxic effects were detected for the lowest studied concentration, but a significant reduction of cell viability (by 20 ± 4%) was observed for the treatment with the higher concentrations. After 48 h of incubation with free ATRA, for all the tested concentrations, the cell viability reduced to 60–50%. This effect suggests a time point when the neuroblastoma cells differentiation begins because the process is characterised by the loss of cell adherence to substratum and apoptosis [81].

After 24 h of incubation with PVA@Rhod or PVA@RhodFR, a minor reduction of cell viability (approximately 6 ± 3% less viable cells than the untreated control) occurs at the lowest polymer concentration, which further decreases at the higher concentrations (approximately 15 ± 4% less viable cells than the untreated control). After 48 h of treatment, a significant cytotoxic effect (cell viability reduction of 45%) was observed, demonstrating the ability of the water-soluble PVA polymer to enter the SH-SY5Y cells, with consequent rhodamine B accumulation [82]. For instance, cationic compounds such as rhodamine B have been demonstrated to accumulate in the mitochondria of tumour cells, which have a highly negative membrane potential compared to normal cells. Upon accumulation and subsequent retention, these cationic compounds can lead to disruption of adenosine triphosphate (ATP) synthesis, causing mitochondrial dysfunction and cell death [83,84]. In the case of the cells treated with the PVA@Rhod + ATRA mixture, for both 24 and 48 h of incubation, the trend profile for the cell viability was very similar to that observed for the free ATRA, i.e., approximately a 20 ± 4% reduction of cell viability for the highest concentration at 24 h and a cell viability reduction of about 30 ± 10% for all concentrations after 48 h. 

Overall, the differences observed between the treatments with PVA@RhodFR samples can be attributed, in agreement with optical and confocal images analysis (Figure 8, Figure 9 and Figure 10), to the combination of different processes involving both the cell differentiation and their detachment from the culture plate, most likely due to the higher bioavailability and biological efficacy of the ATRA covalently linked to the polymer compared with the free ATRA. However, for the PVA@Rhod + ATRA-treated cells, the cytotoxic effect is most likely due to the rhodamine-labelled polymer activity.

## 4. Conclusions

The development of a new multifunctional polymer conjugate based on a PVA scaffold containing FA as a targeting agent, rhodamine B as a tracking agent, and ATRA as the active drug was obtained through a one-pot esterification reaction. 

The PVA was obtained by hydrolysis of synthesised PVAc and subsequently used to develop the multifunctional polymer conjugate, PVA@RhodFR. The rhodamine B, folic acid, and ATRA content in the polymer conjugate were determined as 0.06, 0.15, and 0.40 mol%, respectively.

The chemical–physical characterisation revealed that the polymer conjugate maintains the optical properties (absorbance and fluorescence emission) of the tracking agents and retains high water solubility. The cytotoxic assays confirmed negligible cytotoxicity of the conjugate. 

Differentiation experiments of SH-SY5Y neuroblastoma cells revealed the effective biological action of PVA@RhodFR. The SH-SY5Y cells displayed neuron-like morphology with long neurite and branch outgrowth upon treatment with PVA@RhodFR, even after a short incubation time (t = 5 h). In addition to developing ATRA–polymer conjugates, the approach described here can be exploited to obtain water-soluble conjugates that are useful in theranostics by covalently linking different drugs and targeting and tracking agents to the PVA chain. 

Finally, this work highlights the ability to tailor polymer conjugates, making them suitable candidates for designing affordable individualised therapies. In other words, drugs and treatments can be tailored to the needs of the patient, including their susceptibility, drug resistance, clinical history, disease status, and so on. 

## Data Availability

The data supporting the conclusions are included in the manuscript and Supplementary Materials.

## Supplementary Materials

The following supporting information can be downloaded at: https://www.mdpi.com/article/10.3390/polym14204329/s1, Figure S1: ^1^H-NMR spectrum of PVAc (CDCl_3_); Figure S2: MALDI-TOF mass spectrum of PVAc; Figure S3: ^1^H-NMR spectrum of CoVAc-VA (CDCl_3_); Figure S4: ^1^H-NMR spectrum of PVA (D_2_O).
