# Supplementary material for: A Multifunctional Conjugated Polymer Developed as an Efficient System for Differentiation of SH-SY5Y Tumour Cells
Source: Polymers (Basel). 2022 Oct 14;14(20):4329. doi: 10.3390/polym14204329 (PMC9609355; doi:10.3390/polym14204329)
Supplement: Supplementary file 1 [file polymers-14-04329-s001.zip › polymers-1945676-supplementary.pdf]

Supplementary Materials

A Multifunctional Conjugated Polymer Developed as an Efficient System for Differentiation of SH-SY5Y Tumour Cells

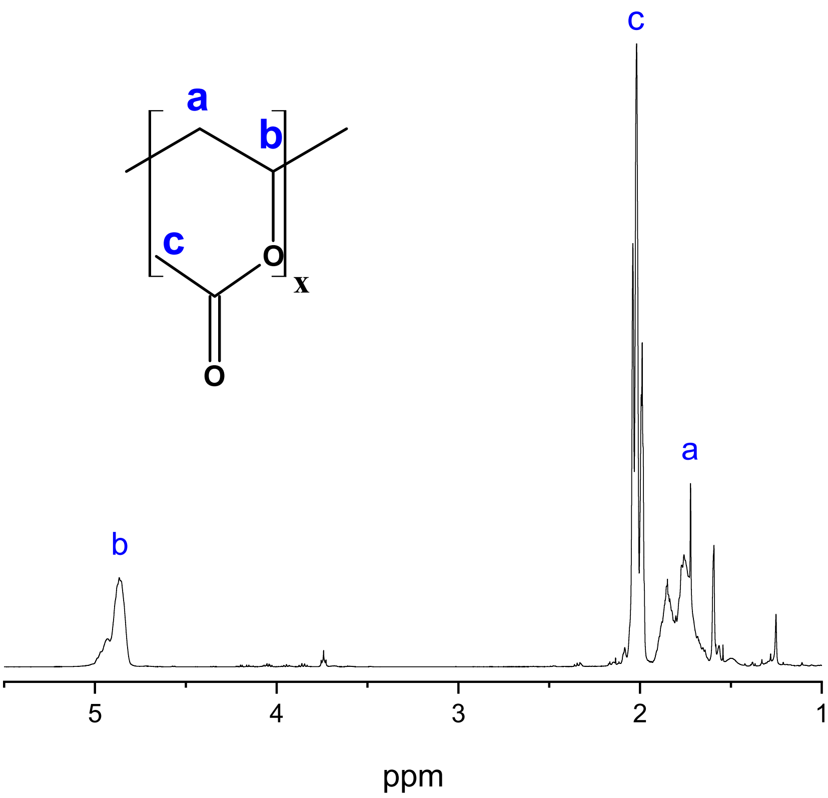

Figure S1. ^{1}H-NMR spectrum of **PVAc** ($CDCl_3$).

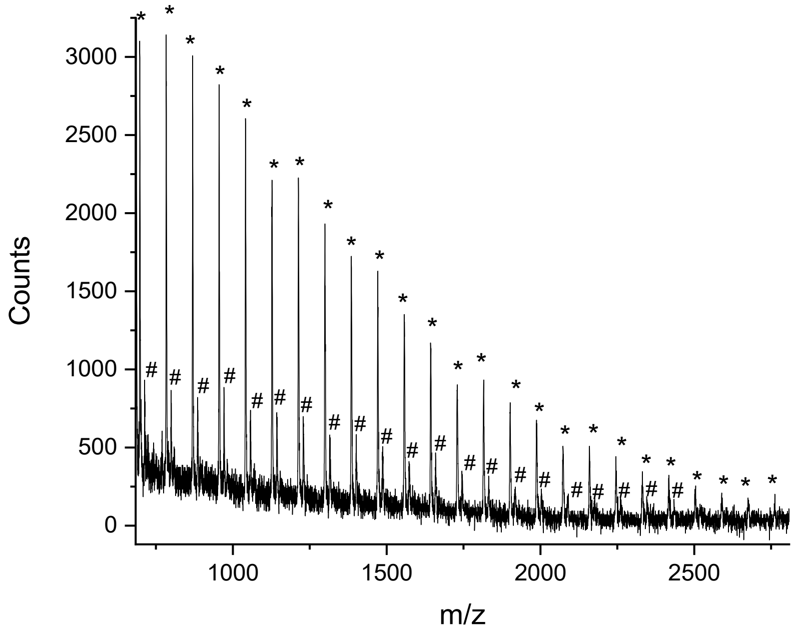

Figure S2. MALDI-TOF mass spectrum of **PVAc**.

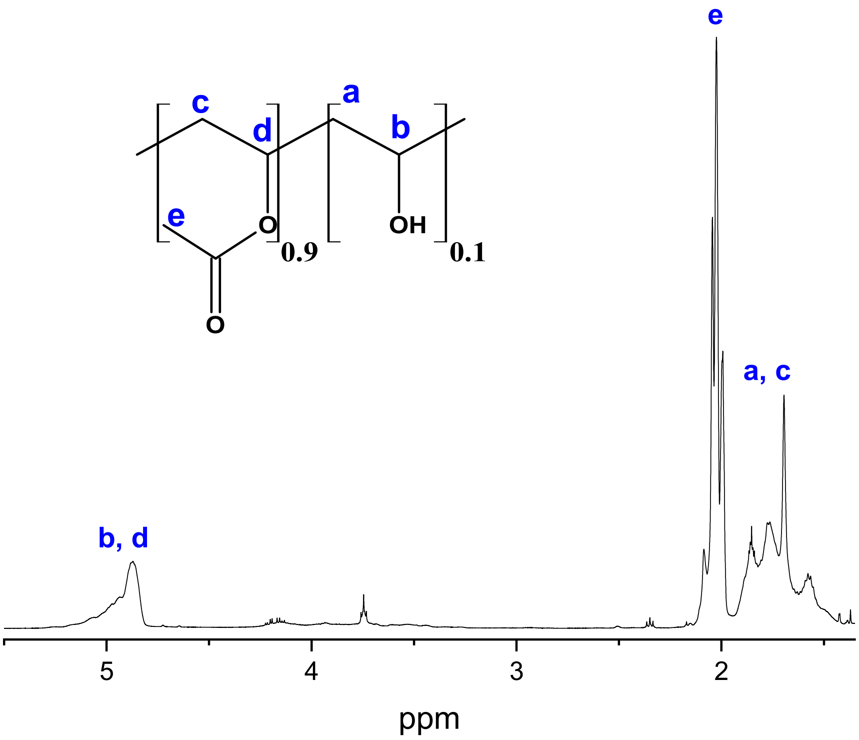

Figure S3. ^{1}H-NMR spectrum of **CoVAc-VA** ($CDCl_3$).

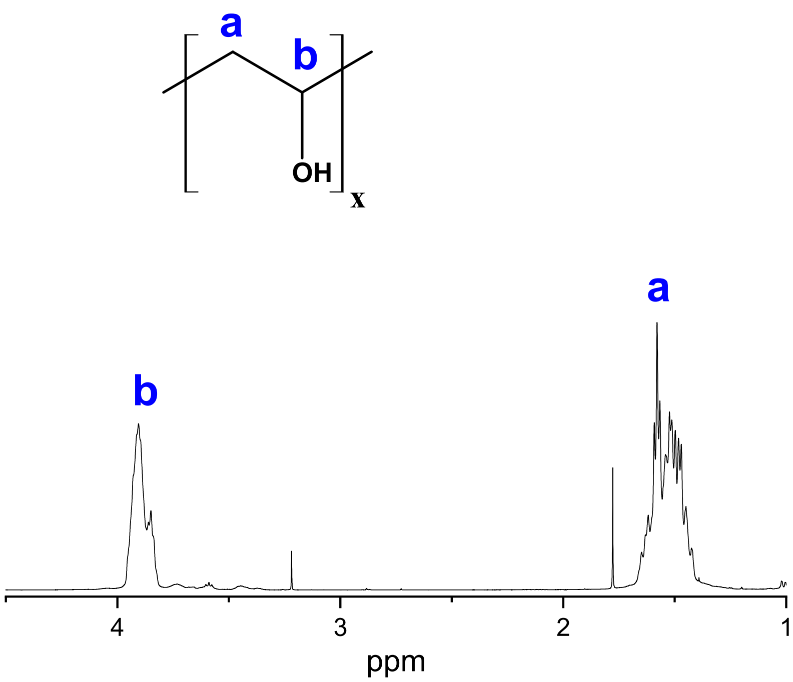

Figure S4. ^{1}H-NMR spectrum of **PVA** (D_2O).
